# Diagnosis, Perioperative Management, and Surgical Treatment of Suspected Functional Pheochromocytoma in Two Dogs: Surgical Complexity in the Absence of Planned Venotomy

**DOI:** 10.3390/vetsci13080749

**Published:** 2026-07-28

**Authors:** Hyungsan Seo, Beom-Joon Park, Yookyeong Lee, Hyung-Seok Seo, Hwi-Yool Kim

**Affiliations:** Department of Veterinary Surgery, College of Veterinary Medicine, Konkuk University, 120, Neungdong-ro, Gwangjin-gu, Seoul 05029, Republic of Korea; shs7683@konkuk.ac.kr (H.S.); park0510k@gmail.com (B.-J.P.); wwwatangerine@gmail.com (Y.L.); gudtjrdldl@konkuk.ac.kr (H.-S.S.)

**Keywords:** canine pheochromocytoma, adrenalectomy, peri-vascular adhesion, vascular distortion, patient-specific surgical planning, three-dimensional vascular reconstruction, computed tomography, adrenal tumor, veterinary surgery, case report

## Abstract

Functional pheochromocytoma is an uncommon canine adrenal tumor associated with substantial surgical and anesthetic challenges. This report describes two dogs with suspected functional pheochromocytoma that exhibited distinct causes of surgical complexity. Both underwent endocrine evaluation, contrast-enhanced CT, adrenalectomy, and histopathology. In Case 1, severe perivascular adhesion resulted in an iatrogenic caudal vena cava laceration despite the absence of definitive vascular invasion, requiring primary venorrhaphy. In Case 2, marked tumor-induced vascular distortion obscured critical anatomical landmarks, necessitating staged surgical management. Three-dimensional vascular reconstruction facilitated refinement of the surgical approach, while individualized anesthetic management enabled successful surgery despite marked intraoperative hemodynamic fluctuations. Histopathology confirmed pheochromocytoma in both dogs, including malignant pheochromocytoma in Case 2. Case 1 developed chronic kidney disease during long-term follow-up, whereas no clinical evidence of local recurrence or distant metastasis was observed during the available follow-up. These cases demonstrate that surgical complexity may arise from severe perivascular adhesion and tumor-induced vascular distortion, even without planned vascular lumen entry or advanced vascular procedures. Patient-specific anatomical assessment, individualized perioperative planning, and selective 3D vascular reconstruction may improve surgical safety in complex adrenal tumors.

## Simple Summary

Pheochromocytoma is an adrenal tumor that often develops in close proximity to major abdominal vessels, making surgical removal particularly challenging. Surgeons usually focus on whether the tumor has invaded major blood vessels when estimating surgical difficulty. However, the two canine cases presented in this report demonstrate that marked operative complexity may arise through different mechanisms even in the absence of planned vascular lumen entry or advanced vascular procedures. In one dog, severe fibrous adhesion between the tumor and the caudal vena cava caused accidental vascular injury despite the absence of macroscopic invasion of the major abdominal vessels. In the other dog, marked distortion of the regional vascular anatomy obscured normal surgical landmarks and prevented safe continuation of the initial surgery. Additional 3D reconstruction of computed tomography images provided patient-specific anatomical information that facilitated refinement of the surgical approach and successful tumor removal. These cases illustrate that vascular adhesion, anatomical distortion, and loss of normal anatomical landmarks may contribute to surgical complexity in dogs with pheochromocytoma and may warrant careful consideration during patient-specific perioperative planning.

## 1. Introduction

Pheochromocytoma is a neuroendocrine tumor originating from chromaffin cells of the adrenal medulla and is characterized by autonomous catecholamine secretion [1,2,3]. In dogs, functional pheochromocytomas may result in systemic hypertension, tachyarrhythmias, and marked perioperative cardiovascular instability [4,5,6]. In addition to their endocrine effects, these tumors are clinically important owing to their propensity for locally aggressive behavior and their intimate anatomical relationships with major retroperitoneal vascular structures [1,7,8].

Surgical excision remains the treatment of choice for canine pheochromocytoma [2,8,9]. However, adrenalectomy is frequently challenging because of the close anatomical association between the adrenal glands and major abdominal vessels [8,9,10]. Traditionally, right-sided adrenalectomy is considered more technically demanding because of the proximity of the right adrenal gland to the caudal vena cava (CdVC), hepatic vasculature, and phrenicoabdominal vein [8,9,10]. Consequently, dense peri-vascular adhesions, vascular invasion, and major intraoperative hemorrhage are well-recognized challenges during surgical management of right-sided adrenal masses [7,11,12]. Even in the absence of definitive intraluminal vascular invasion, extensive peri-vascular fibrosis and adhesion may substantially increase operative difficulty and predispose patients to catastrophic vascular injury during tumor dissection [7,8]. Accordingly, much of the existing veterinary literature has focused on surgical techniques for adrenal tumors with major vascular invasion requiring venotomy, thrombectomy, or vascular reconstruction [13,14,15]. In contrast, comparatively little attention has been paid to cases in which marked surgical complexity arises despite the absence of vascular lumen entry or the need for advanced vascular procedures.

Although left-sided adrenalectomy is generally regarded as technically less demanding than right-sided adrenalectomy, progressive enlargement of adrenal masses may markedly distort the normal retroperitoneal anatomy [8,9]. Tumor growth can alter the course and morphology of adjacent vessels, resulting in displacement of the phrenicoabdominal vein, left renal vessels, and major visceral arteries [8,10,15]. Under these circumstances, conventional anatomical landmarks may become unreliable, making differentiation between expendable vessels and major visceral arteries increasingly difficult [9,10]. In particular, severe distortion of the phrenicoabdominal vein may obscure its normal anatomical relationship with adjacent vascular structures and complicate safe dissection around the celiac and cranial mesenteric arteries (CMA) [10,15]. Therefore, surgical complexity may arise not only from vascular invasion itself but also from profound tumor-associated anatomical distortion that obscures the true relationships among adjacent vascular structures.

Accurate preoperative assessment should therefore extend beyond evaluation of vascular involvement alone and include careful characterization of tumor-induced vascular displacement and distorted vascular anatomy [16,17]. Contrast-enhanced computed tomography (CT) is widely utilized for staging adrenal masses and assessing vascular involvement [16,17]. However, conventional two-dimensional CT images may not fully reflect the complexity of the operative field or the degree of vascular displacement encountered during surgery [17,18]. As tumor size increases, substantial discrepancies may arise between preoperative imaging findings and actual intraoperative anatomy, particularly when major vascular structures are displaced, compressed, or obscured by the mass [16,19].

To overcome these limitations, advanced three-dimensional (3D) vascular reconstruction techniques have increasingly been adopted to improve visualization of tumor–vessel relationships and facilitate surgical planning [18,20]. 3D vascular reconstruction provides intuitive visualization of distorted vascular anatomy, enabling surgeons to identify critical vascular structures, select an appropriate surgical approach, and anticipate potential intraoperative challenges [18,21]. Such information may be particularly valuable when conventional anatomical landmarks are lost because of severe vascular distortion [18,20,21]. Nevertheless, reports describing the application of advanced 3D vascular reconstruction for canine pheochromocytomas associated with severe anatomical distortion remain limited [2,20].

In addition to surgical complexity, perioperative anesthetic management represents a critical component of successful treatment [6,22]. Despite preoperative α-adrenergic blockade, substantial cardiovascular fluctuations may occur during manipulation of functional pheochromocytomas, necessitating tailored anesthetic management to maintain hemodynamic stability throughout surgery [6,22,23].

This report describes two dogs with suspected functional pheochromocytoma that presented distinct yet equally challenging surgical scenarios. In one dog, extensive peri-vascular adhesion resulted in intraoperative CdVC injury requiring immediate vascular repair despite the absence of definitive caval invasion on preoperative imaging. In the second dog, severe tumor-associated vascular distortion obscured critical anatomical landmarks and prevented safe identification of major visceral arteries, necessitating repeat imaging with advanced 3D vascular reconstruction, modification of the surgical approach, and tailored anesthetic management. Importantly, neither case ultimately required venotomy or thrombectomy despite substantial surgical complexity, illustrating that operative difficulty may arise from mechanisms other than direct vascular invasion alone. These cases highlight the importance of individualized perioperative planning based not only on vascular invasion but also on the degree of tumor-associated vascular distortion and loss of normal anatomical landmarks. Throughout this report, vascular invasion refers to macroscopic invasion of major named vessels identified on preoperative imaging or during surgery that would influence operative planning or require advanced vascular procedures. This definition does not include microscopic vascular invasion identified only on histopathological examination.

## 2. Case Description

### 2.1. Signalment, Clinical Presentation, and Endocrinological Evaluation

Two client-owned dogs—a 12-year-old castrated male Bichon Frise weighing 8.5 kg (Case 1) and a 6-year-old castrated male Maltese weighing 3.0 kg (Case 2)—were referred to our hospital following the incidental detection of adrenal masses during abdominal ultrasonography performed at local veterinary hospitals. The masses originated from the right adrenal gland in Case 1 and the left adrenal gland in Case 2.

On presentation, both dogs were bright, alert, and responsive and exhibited no overt clinical signs suggestive of catecholamine excess. However, elevated systolic arterial pressure (SAP) was identified in both patients. SAP was measured using a Doppler sphygmomanometer. Five consecutive measurements were obtained at each assessment, with the first and last measurements discarded and the mean of the remaining three values recorded. In Case 1, the initial mean SAP was 183 mmHg and remained elevated at 180 mmHg after 30 min of acclimatization in a quiet environment, consistent with persistent systemic hypertension. In Case 2, SAP was measured once daily on four consecutive days during hospitalization using the same protocol, before initiation of medical treatment. The mean SAP values were 150, 160, 190, and 170 mmHg. The repeated elevations to 170 and 190 mmHg were considered suggestive of intermittent systemic hypertension, although SAP was not consistently above the conventional diagnostic threshold.

To differentiate suspected functional pheochromocytoma from cortisol-secreting adrenocortical tumors, endocrine evaluation was performed individually in each patient. In Case 1, a urine corticoid-to-creatinine ratio (UCCR) was within the reference interval (16.9%; reference interval, 10.1–33.9%), excluding hypercortisolism. In Case 2, an adrenocorticotropic hormone (ACTH) stimulation test performed at the referring hospital yielded an equivocal result (pre-ACTH cortisol, 2.5 μg/dL; post-ACTH cortisol, 20.7 μg/dL), suggesting possible hyperadrenocorticism. However, a subsequent low-dose dexamethasone suppression test (0 h, 2.5 μg/dL; 4 h, <0.5 μg/dL; 8 h, <0.5 μg/dL) excluded hypercortisolism [24].

Subsequently, urinary normetanephrine-to-creatinine ratio (NMN:Cr) was measured by liquid chromatography–tandem mass spectrometry (LC-MS/MS) at a commercial veterinary diagnostic laboratory (GreenVet, Yongin, Republic of Korea). The NMN:Cr was markedly increased in both dogs (Case 1, 616.6 U; Case 2, 1697 U; laboratory reference interval, 14.0–91.0 U [25]), supporting the diagnosis of suspected functional pheochromocytoma. Cardiac auscultation revealed no audible murmur in either dog, and thoracic radiographs demonstrated no evidence of clinically significant cardiac enlargement. Therefore, additional preoperative cardiac evaluation, including echocardiography, was not performed. Combined with persistent systemic hypertension, these findings supported the diagnosis of suspected functional pheochromocytoma, prompting further diagnostic imaging and surgical planning.

### 2.2. Diagnostic Imaging

Both patients underwent comprehensive preoperative imaging, including transabdominal ultrasonography and contrast-enhanced CT, to characterize the adrenal masses and evaluate their relationships with adjacent vascular structures.

**Figure 1:**
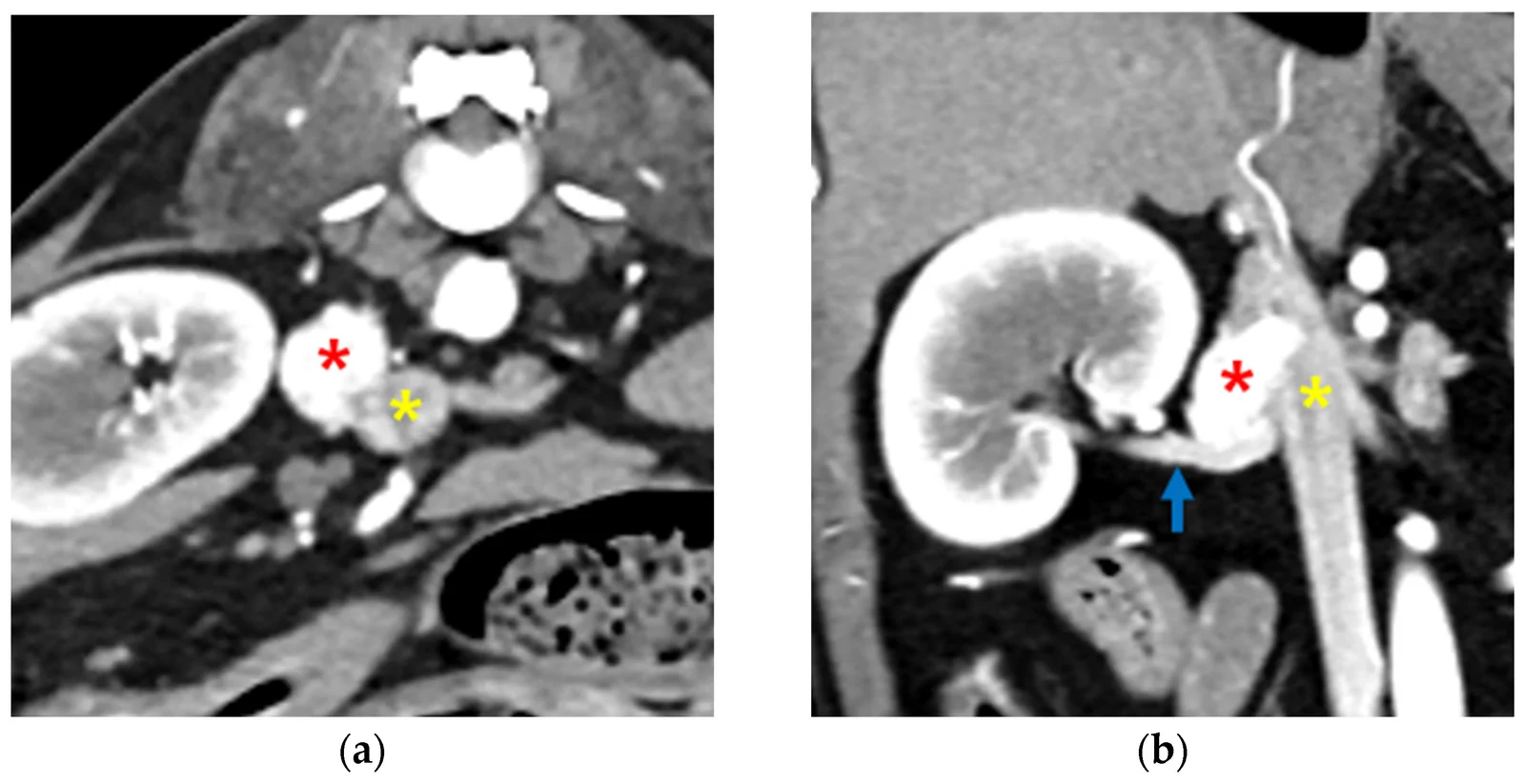
Preoperative contrast-enhanced computed tomography images of Case 1. (**a**) Transverse and (**b**) coronal computed tomography images demonstrating a right adrenal mass (red asterisk) closely associated with the caudal vena cava (yellow asterisk) and right renal vein (blue arrow). Loss of the normal fat plane between the mass and the right renal vein raised suspicion of vascular involvement.

Case 1: Transabdominal ultrasonography revealed a large, heteroechoic, irregularly shaped mass arising from the right adrenal gland. Contrast-enhanced CT identified a well-defined right adrenal mass measuring 3.2 × 2.8 × 2.5 cm. The mass displaced the descending duodenum and right kidney leftward and was closely associated with several major retroperitoneal vessels. Loss of the normal fat plane and an indistinct interface between the mass and both the CdVC and right renal vein raised suspicion of subtle vascular invasion (Figure 1a,b). The mass also closely contacted the right renal artery and adjacent lumbar vessels. However, no definitive intraluminal tumor thrombus, complete vascular occlusion, or other imaging findings indicating extensive vascular invasion were identified. Preoperative renal function was considered adequate to permit unilateral ureteronephrectomy, with a blood urea nitrogen (BUN) concentration of 11 mg/dL and a serum creatinine concentration of 0.8 mg/dL.

Case 2: Transabdominal ultrasonography identified a large left adrenal mass associated with marked distortion of the surrounding retroperitoneal anatomy. Initial contrast-enhanced CT performed at the referring hospital demonstrated a heterogeneously enhancing left adrenal mass measuring approximately 2.9 × 2.4 × 2.1 cm. Although no definitive vascular involvement or tumor thrombus was identified, the mass exerted substantial mass effect on the surrounding vasculature, resulting in severe displacement and deformation of adjacent vessels.

In particular, the normal orientation of the phrenicoabdominal vein was markedly altered, making its anatomical relationship with neighboring vascular structures difficult to appreciate using conventional multiplanar CT images. Consequently, reliable preoperative differentiation between the phrenicoabdominal vein, left renal vessels, celiac artery, and CMA could not be confidently established. Because preservation of these major visceral arteries was considered essential for safe adrenalectomy, complete characterization of the distorted vascular anatomy remained challenging based on the initial CT examination alone.

**Figure 2:**
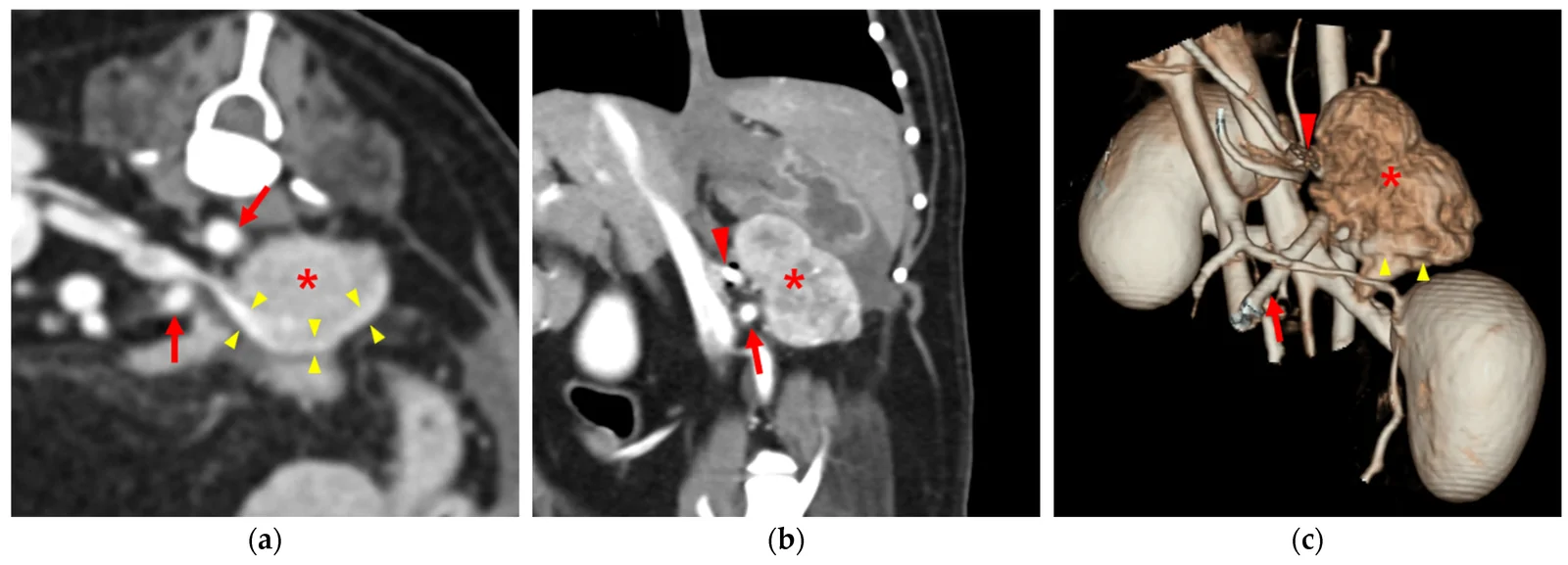
Repeat contrast-enhanced computed tomography and three-dimensional vascular reconstruction in Case 2. The adrenal mass (red asterisk), cranial mesenteric artery (red arrow), phrenicoabdominal vein (yellow arrowhead), and celiac artery (red arrowhead) are indicated in all panels. (**a**) Transverse computed tomography image demonstrating the close spatial relationship between the cranial mesenteric artery and the phrenicoabdominal vein adjacent to the adrenal mass. (**b**) Coronal computed tomography image demonstrating the close proximity of the cranial mesenteric artery and celiac artery to the adrenal mass. (**c**) Three-dimensional volume-rendered reconstruction demonstrating the anatomical relationships between the adrenal mass and surrounding vasculature.

After referral to our institution, exploratory laparotomy was performed. However, because the distorted vascular anatomy prevented reliable identification of critical vascular structures, the procedure was intentionally discontinued before adrenalectomy could be completed, and the abdomen was routinely closed. Repeat contrast-enhanced CT was performed immediately after abdominal closure using a 64-slice multidetector CT scanner (Aquilion Lightning; Canon Medical Systems, Otawara, Japan) with a slice thickness of 1 mm (120 kVp, 112 mAs). Iohexol (Omnipaque^®^, GE Healthcare, Anaheim, CA, USA) was administered intravenously at 3 mL/kg using a power injector at a rate of 2.0 mL/s. Arterial, portal venous, and delayed phase images were acquired. 3D volume-rendered vascular reconstruction was subsequently generated on a dedicated workstation using Vitrea software (Canon Medical Systems, Otawara, Japan) (Figure 2). Based on these findings, definitive adrenalectomy was performed 5 days after the initial exploratory laparotomy.

The volume-rendered reconstructed images clearly delineated the altered course of the phrenicoabdominal vein, CMA, celiac artery, and left renal vessels relative to the adrenal mass, providing a comprehensive anatomical roadmap for definitive surgical planning.

### 2.3. Preoperative Medical Management and Anesthetic Protocols

For preoperative management of the suspected functional pheochromocytomas, oral phenoxybenzamine was administered to both patients to reduce catecholamine-associated cardiovascular risk. Case 1 received phenoxybenzamine at 0.5 mg/kg PO every 12 h for 2 weeks, resulting in a reduction in SAP from approximately 180 mmHg to 150 mmHg before surgery. In contrast, after two weeks of the same treatment protocol, preoperative SAP remained approximately 180 mmHg. Because SAP remained relatively stable without evidence of uncontrolled hypertensive crises, further dose escalation was not performed. Preoperative α-adrenergic blockade was considered clinically adequate based on overall hemodynamic stability, and additional phenoxybenzamine administration was considered to carry a potential risk of excessive hypotension following tumor removal.

On the day of surgery, both dogs received a standardized anesthetic protocol. Arterial blood pressure was monitored non-invasively using an oscillometric monitor throughout anesthesia, and SAP and mean arterial pressures (MAP) were recorded. For the purposes of intraoperative assessment, bradycardia was defined as a heart rate below 60 beats/min. Clinically significant bradycardia was defined as bradycardia requiring interruption of surgical manipulation or modification of the anesthetic protocol. Premedication consisted of midazolam (0.3 mg/kg IV; Bukwang Midazolam Inj., Bukwang Pharm, Seoul, Republic of Korea). General anesthesia was induced with propofol (up to 5 mg/kg IV; Anepol Inj., Hana Pharm, Seoul, Republic of Korea) and maintained with isoflurane (Terrell Solution, Kyongbo Pharm, Asan, Republic of Korea) in 100% oxygen. Amoxicillin–clavulanic acid (13.75 mg/kg IV; Amocra Inj., Kuhnil Pharm, Cheonan, Republic of Korea) and famotidine (0.5 mg/kg IV; Gaster Injection, Dong-A ST, Seoul, Republic of Korea) were administered at anesthetic induction, and amoxicillin–clavulanic acid was re-administered every 90 min throughout the procedure.

Case 1: Analgesia was achieved using fentanyl (3 μg/kg IV bolus) followed by a fentanyl continuous-rate infusion (CRI; 2–10 μg/kg/h) throughout adrenalectomy. No clinically apparent anesthetic complications or hemodynamic instability requiring additional anesthetic intervention were observed during the procedure.

Case 2: During the initial exploratory laparotomy performed at our institution, analgesia was provided using fentanyl (3 μg/kg IV bolus followed by a fentanyl CRI of 2–10 μg/kg/h) in combination with dexmedetomidine (0.25–1.0 μg/kg/h CRI; Dorbene Inj., Dongbang Co., Seoul, Republic of Korea) and maropitant (0.1 mg/kg/h CRI; Cerenia Inj., Zoetis, Parsippany, NJ, USA). During repeated manipulation of the adrenal mass and adjacent distorted vascular structures, recurrent episodes of marked bradycardia were observed, with heart rate decreasing to approximately 50 beats/min. These episodes repeatedly interrupted surgical manipulation and, together with the inability to safely identify the distorted vascular anatomy, ultimately resulted in discontinuation of the procedure.

For the definitive adrenalectomy performed five days after the abandoned exploratory laparotomy, dexmedetomidine and maropitant CRIs were intentionally maintained because suppression of sympathetic activation and catecholamine release during adrenal manipulation was considered more important than eliminating a potential contributor to bradycardia in the presence of persistent systemic hypertension despite preoperative phenoxybenzamine administration. Because dexmedetomidine was intentionally maintained, reduction in opioid administration rather than withdrawal of dexmedetomidine was selected as the primary strategy to minimize recurrent bradycardia. Accordingly, fentanyl was replaced with remifentanil (0.1–0.3 μg/kg/min CRI; Remiva Inj., Hana Pharm, Seoul, Republic of Korea) to permit rapid titration of opioid administration during periods of abrupt intraoperative hemodynamic change.

Following implementation of the revised anesthetic protocol, recurrent episodes of clinically significant bradycardia did not recur despite repeated manipulation of the adrenal mass and adjacent major vascular structures. Although the lowest recorded heart rate was 59 beats/min, these transient decreases did not require interruption of surgery or additional therapeutic intervention and therefore were not considered clinically significant. During definitive adrenalectomy, SAP ranged from 83 to 217 mmHg, mean arterial pressure ranged from 60 to 165 mmHg, and heart rate ranged from 59 to 208 beats/min. Whenever tumor manipulation was considered to induce a catecholamine surge with acute hypertension and tachycardia, the dexmedetomidine CRI was temporarily increased. After stabilization of blood pressure and heart rate, the infusion rate was gradually reduced to the maintenance dose. Hemodynamic instability resolved within approximately 5 min following these adjustments, allowing continuous surgical dissection and completion of the procedure. Because cardiovascular stability was restored promptly, additional vasoactive agents, including phentolamine, esmolol, or nitroprusside, were not required.

### 2.4. Surgical Intervention and Postoperative Outcome

**Figure 3:**
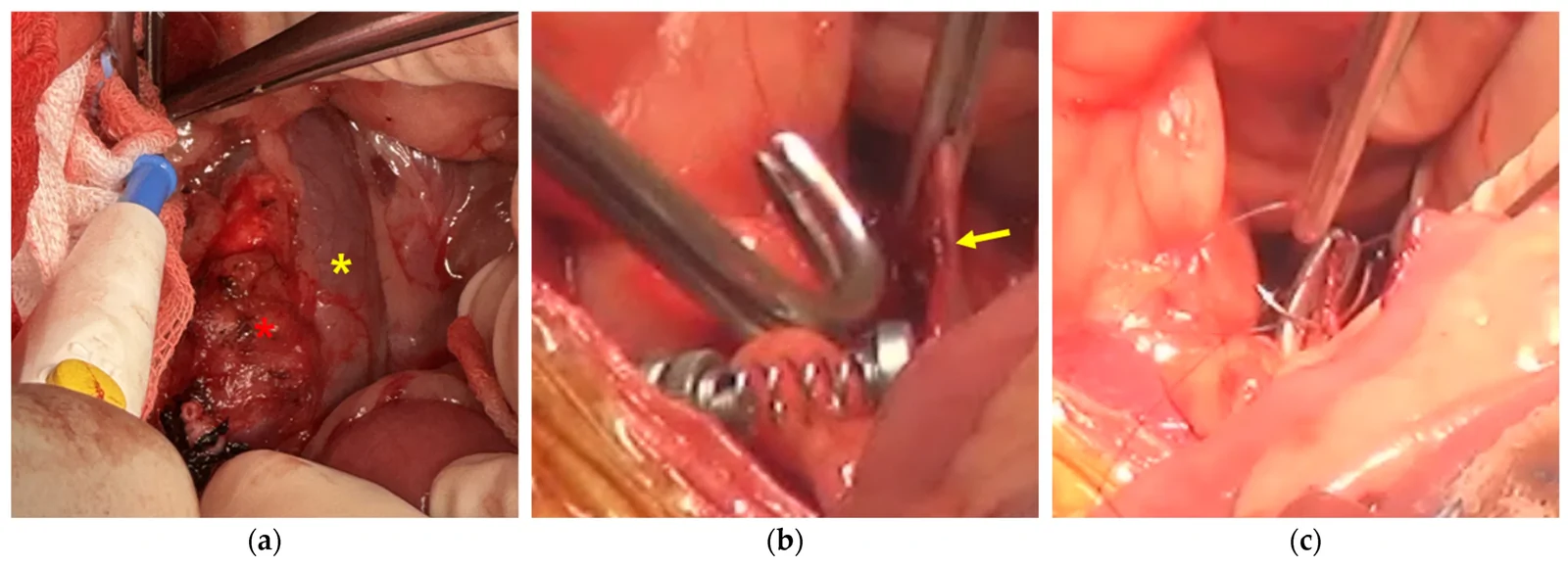
Intraoperative findings and vascular repair in Case 1. The adrenal mass (red asterisk), caudal vena cava (yellow asterisk), and caval laceration site (yellow arrow) are indicated. (**a**) Intraoperative view demonstrating the close association between the adrenal mass and the caudal vena cava following dissection of the surrounding tissues. (**b**) Intraoperative image showing the iatrogenic laceration of the caudal vena cava during separation of the mass from the vessel wall. (**c**) Primary venorrhaphy of the caudal vena cava following vascular control and hemostasis.

Case 1: The patient was positioned in dorsal recumbency, and the right retroperitoneal space was approached through a standard ventral midline celiotomy. Surgical exploration revealed a large right adrenal mass that was firmly adherent to the right renal vein (Figure 3a). Because separation of the tumor from the right renal vein was considered unlikely to achieve complete excision with an adequate surgical margin and was judged to carry an increased risk of intraoperative hemorrhage, a right ureteronephrectomy was performed. The right renal artery, right renal vein, and ureter were individually isolated, ligated using 2-0 black silk, and transected.

Following ureteronephrectomy, the phrenicoabdominal vein was identified, ligated, and transected to facilitate exposure of the adrenal mass. Blunt dissection was then performed to separate the medial aspect of the tumor capsule from the wall of the CdVC, where extensive fibrous adhesion was encountered. During careful dissection of the adhesion plane using cotton-tipped applicators, an iatrogenic longitudinal laceration developed in the ventral wall of the CdVC, resulting in acute hemorrhage.

Hemorrhage was immediately controlled using a Satinsky vascular clamp. Temporary vascular occlusion was established using a bulldog clamp on the caudal aspect of the CdVC and DeBakey forceps on the cranial aspect of the vessel (Figure 3b). Primary venorrhaphy was successfully performed using 6-0 polydioxanone suture in a continuous pattern (Figure 3c). Following restoration of vascular integrity and confirmation of complete hemostasis, the remaining soft tissue attachments were dissected and complete adrenalectomy was achieved. A total of 100 mL of compatible whole blood was administered intraoperatively and during the immediate postoperative period to compensate for blood loss. The preoperative packed cell volume (PCV) was 34.2%, and the PCV measured after surgery and blood transfusion was 31.4%. Following completion of the venorrhaphy and nephrectomy, complete hemostasis was confirmed, and routine abdominal closure was performed.

**Figure 4:**
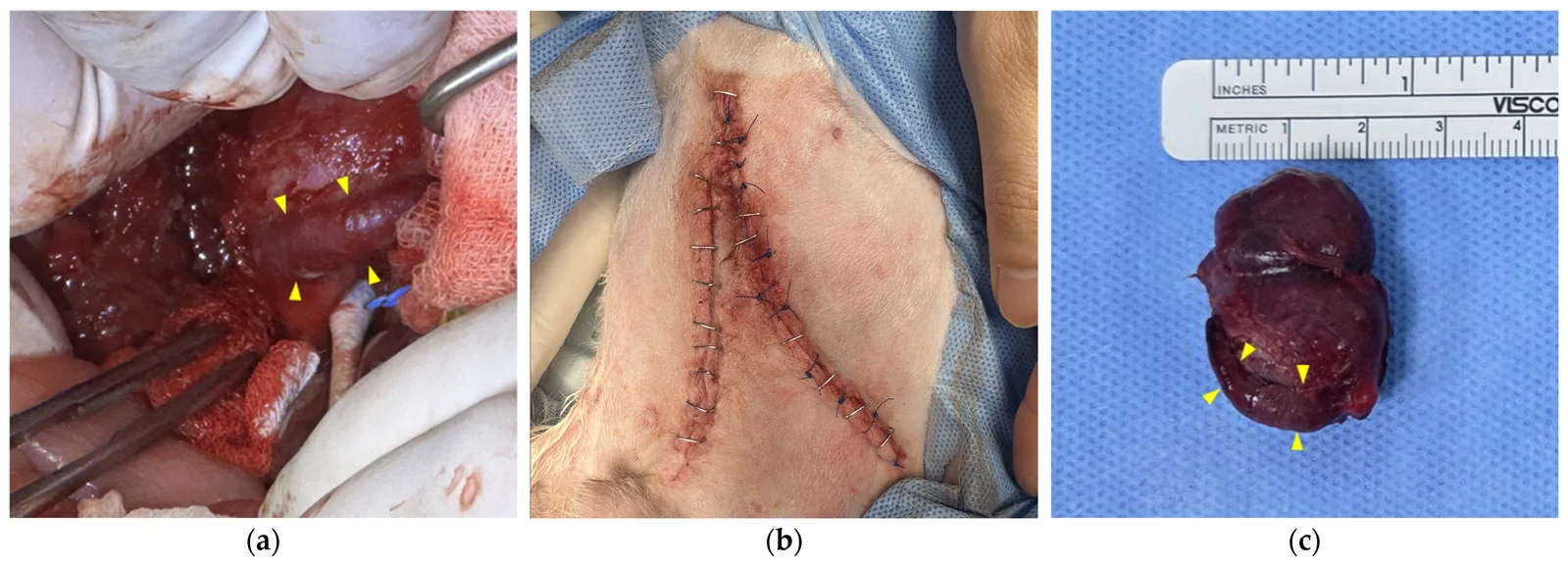
Intraoperative findings and gross appearance of the resected adrenal mass in Case 2. The phrenicoabdominal vein (yellow arrowheads) is indicated. (**a**) Intraoperative image demonstrating the distorted phrenicoabdominal vein adjacent to the adrenal mass following surgical exposure of the left retroperitoneal space. (**b**) Final appearance of the surgical field following routine abdominal closure with a ventral midline incision and an additional left paracostal incision. (**c**) Gross specimen of the excised adrenal mass with the attached phrenicoabdominal vein.

Case 2: During the initial exploratory laparotomy performed at our institution, the dog was positioned in dorsal recumbency, and a routine ventral midline celiotomy was performed. Severe distortion of the regional vascular anatomy was encountered (Figure 4a). The enlarged adrenal mass displaced multiple major vascular structures and markedly altered the normal orientation of the phrenicoabdominal vein, preventing its reliable use as an anatomical landmark for adrenalectomy. Consequently, the distorted vascular anatomy precluded confident differentiation between the tumor margin and adjacent major visceral arteries, including the celiac artery, CMA, and left renal vessels. Because continuation of the procedure was considered to carry an unacceptable risk of catastrophic vascular injury, the operation was intentionally discontinued and the abdomen was routinely closed.

Five days after the abandoned exploratory laparotomy, definitive adrenalectomy was performed using the surgical plan established from repeat contrast-enhanced CT and 3D reconstruction. The dog was positioned in a slightly oblique dorsal recumbency to facilitate extension of the surgical field toward the left cranial abdomen. The original ventral midline incision was extended with a left paracostal incision to improve surgical exposure (Figure 4b). The reconstructed images enabled reliable preoperative identification of the displaced phrenicoabdominal vein, celiac artery, CMA, and left renal vessels, thereby restoring anatomical orientation before surgical dissection.

Guided by these preoperative findings, the major vascular structures were successfully identified and distinguished from the tumor margins before adrenal dissection was initiated. Complete adrenalectomy was subsequently accomplished without major vascular injury, uncontrollable hemorrhage, venotomy, or vascular reconstruction (Figure 4c). Following confirmation of complete hemostasis, routine abdominal closure was performed.

Histopathological examination confirmed pheochromocytoma in both dogs. In Case 1, the adrenal mass was completely excised with narrow but histologically complete surgical margins (<1 mm). The mitotic count was 2 per 10 high-power fields, and no vascular invasion was identified within the submitted adrenal specimen. Immunohistochemistry was not performed because the diagnosis was considered definitive based on routine histopathological examination. Only the adrenal mass was submitted for histopathological examination. The ipsilateral kidney and renal vein, which were resected en bloc during ureteronephrectomy, were not submitted for histopathological evaluation. Consequently, although no vascular invasion was identified within the submitted adrenal specimen, direct histological assessment of vascular wall invasion in the resected renal vein could not be performed. In Case 2, histopathological examination confirmed malignant pheochromocytoma characterized by invasion into the surrounding connective tissue and the presence of tumor emboli. The histopathology report did not include assessment of surgical margin status or a standardized mitotic count. Immunohistochemistry was likewise not performed because the histomorphological findings were considered diagnostic.

Postoperative management focused on cardiovascular stabilization, maintenance of fluid balance, and prevention of thromboembolic complications. Postoperatively, amoxicillin–clavulanic acid (13.75 mg/kg IV every 12 h) was administered for 10 days in both patients. Dalteparin (100 IU/kg SC every 8 h) was administered throughout hospitalization until discharge for postoperative thromboprophylaxis. This dosage was selected because dogs undergoing adrenalectomy for pheochromocytoma are considered at increased risk of thromboembolic complications and this is consistent with current veterinary consensus recommendations for low-molecular-weight heparin administration [26]. Serial D-dimer measurements were performed to monitor the patient’s hypercoagulable status during anticoagulant therapy. Anti-factor Xa activity was not measured. Early ambulation and assisted walking were initiated every 3 h beginning in the immediate postoperative period. Both patients recovered uneventfully without major postoperative complications, including pancreatitis, disseminated intravascular coagulation, or clinically significant thromboembolic events, and were discharged in good clinical condition. In Case 1, the dog was discharged in good clinical condition on postoperative day 6. Renal function was monitored throughout hospitalization. Serum BUN and creatinine gradually increased during the postoperative period, reaching 30 mg/dL and 1.3 mg/dL, respectively, at discharge. The dog has remained under long-term follow-up by the internal medicine service. In Case 2, the dog remained hospitalized for 5 days postoperatively and was subsequently transferred to a local veterinary hospital at the owner’s request for continued postoperative care. At the most recent evaluation (postoperative day 645), the BUN and serum creatinine concentrations were 45 mg/dL and 2.8 mg/dL, respectively, and medical management for chronic kidney disease is ongoing. At the time of discharge (Case 1) or transfer to the referring local veterinary hospital (Case 2), SAP had decreased to 130 mmHg and 133 mmHg, respectively. Case 1 has remained under long-term follow-up for 645 postoperative days, and Case 2 for 93 postoperative days. During the available follow-up period, the owners reported no clinical signs suggestive of local recurrence or distant metastasis. Post-discharge follow-up was performed by telephone; therefore, blood pressure measurements and imaging surveillance after discharge were not available.

## 3. Discussion

Successful management of canine pheochromocytoma requires careful integration of preoperative medical stabilization, individualized anesthetic management, detailed anatomical assessment, and meticulous surgical planning [2,8,9]. The two cases presented herein illustrate distinct mechanisms contributing to surgical complexity despite a common diagnosis of suspected functional pheochromocytoma. In Case 1, operative difficulty arose primarily from extensive peri-vascular adhesion despite the absence of definitive caval invasion on preoperative imaging. Although no planned venotomy was required for tumor removal, inadvertent entry into the CdVC occurred following an iatrogenic vascular laceration during tumor dissection and was successfully managed by primary venorrhaphy. In contrast, Case 2 demonstrated that profound tumor-induced vascular distortion alone may substantially complicate adrenalectomy by obscuring normal anatomical landmarks without direct vascular invasion. Together, these cases emphasize that surgical complexity may be evaluated not only on the basis of vascular invasion but also according to the degree of peri-vascular adhesion, vascular displacement, and loss of normal anatomical orientation [7,8,16].

Preoperative α-adrenergic blockade with phenoxybenzamine remains the cornerstone of perioperative management for dogs with functional pheochromocytoma [2,3,6]. Its principal objectives are to attenuate catecholamine-mediated vasoconstriction, restore intravascular volume, and reduce perioperative cardiovascular instability [2,6,22]. Different clinical responses to phenoxybenzamine were observed despite an identical treatment protocol. Variable responses to phenoxybenzamine have previously been described and likely reflect differences in tumor biology, catecholamine secretion, receptor sensitivity, and individual pharmacodynamic variability [2,6]. These findings reinforce that although preoperative α-adrenergic blockade remains indispensable, individualized perioperative management is frequently required to address persistent cardiovascular instability [6,23]. Persistent hypertension in Case 2, despite clinically adequate preoperative α-adrenergic blockade, required additional individualized perioperative anesthetic management beyond conventional phenoxybenzamine administration. In the present cases, adequate preoperative α-adrenergic blockade was judged according to overall hemodynamic stability rather than complete normalization of SAP. Accordingly, although SAP remained higher in Case 2 than in Case 1, further phenoxybenzamine dose escalation was not performed because uncontrolled hypertensive crises were not observed and excessive postoperative hypotension was considered a greater potential risk following adrenalectomy.

Perioperative anesthetic management represented an additional challenge because clinically significant cardiovascular instability occurred during surgical manipulation. Although the precise mechanism could not be definitively established, the observed hemodynamic changes were likely multifactorial and may have reflected the combined effects of opioid administration, dexmedetomidine, catecholamine release associated with tumor manipulation, and autonomic reflexes induced by traction or compression of the severely distorted major visceral vessels [23,27,28]. For the secondary definitive procedure, the anesthetic protocol was modified while maintaining dexmedetomidine because of persistent hypertension despite phenoxybenzamine administration. Fentanyl was replaced with remifentanil to permit more rapid titration of opioid administration during periods of abrupt cardiovascular fluctuation [27,29]. Although dexmedetomidine may increase peripheral vascular resistance through activation of peripheral α2B-adrenoceptors, we considered that, at the low infusion rates used in this case, its central sympatholytic effects predominated. This strategy was intended to blunt sympathetic activation associated with tumor manipulation rather than to directly treat hypertension. Therefore, temporary increases in the dexmedetomidine CRI were intended to attenuate catecholamine release associated with tumor manipulation rather than to directly reduce arterial blood pressure. This modification was not intended solely to address dexmedetomidine-associated bradycardia, but also to facilitate prompt adjustment of opioid administration in response to unpredictable hemodynamic changes that could arise from catecholamine release during tumor manipulation or autonomic reflexes associated with traction of severely displaced major visceral vessels [27,29]. Although conclusions cannot be drawn from a single case, this experience suggests that individualized anesthetic management and selection of readily titratable anesthetic agents may facilitate safer perioperative management during technically demanding adrenalectomy [23,27]. Transient decreases in heart rate below 60 beats/min that resolved spontaneously without interruption of surgery or anesthetic intervention were not considered clinically significant.

Case 1 illustrates that surgical decision-making in canine adrenalectomy should be individualized according to the anticipated relationship between the tumor and adjacent vascular structures rather than based solely on the presence or absence of definitive vascular invasion [2,8,9]. In the present case, ureteronephrectomy was intentionally performed because complete tumor excision with preservation of the right kidney was considered unlikely to achieve an adequate surgical margin and was judged to increase the risk of intraoperative hemorrhage. In contrast, venotomy was not planned because preoperative imaging did not demonstrate definitive caval invasion or intraluminal tumor extension. Nevertheless, dense peri-vascular fibrous adhesion between the tumor capsule and the CdVC complicated dissection and resulted in an iatrogenic caval laceration requiring immediate venorrhaphy. These findings indicate that substantial operative difficulty may arise despite the absence of an indication for planned vascular lumen entry and highlight peri-vascular fibrous adhesion as an additional determinant of surgical complexity that may not be fully appreciated during preoperative imaging [7,16,17].

An important long-term observation in Case 1 was the gradual deterioration of renal function following unilateral ureteronephrectomy. Although preoperative renal function was within the reference interval and the dog recovered uneventfully from surgery, serum creatinine increased progressively during long-term follow-up, and chronic kidney disease was subsequently managed medically. In the present case, ureteronephrectomy was considered necessary because preservation of the ipsilateral kidney was unlikely to permit complete tumor excision and was judged to substantially increase the risk of intraoperative hemorrhage. These findings suggest that the surgical decision remained appropriate based on the intraoperative findings. Nevertheless, the long-term renal outcome emphasizes that nephron loss associated with unilateral nephrectomy may have clinically relevant consequences, even in dogs with apparently normal preoperative renal function [30]. Accordingly, careful preoperative assessment of the contralateral kidney and consideration of long-term renal reserve may be valuable when nephrectomy is anticipated during adrenalectomy.

Contrary to Case 1, although microscopic vascular invasion in the form of tumor emboli was identified histologically after adrenalectomy, no macroscopic invasion of the major named vessels was identified on preoperative imaging or during surgery. Accordingly, the principal intraoperative challenge was profound tumor-induced vascular distortion rather than macroscopic invasion of the major named vessels. Although left-sided adrenalectomy is generally considered technically less demanding than right-sided adrenalectomy because of its greater distance from the CdVC and more straightforward anatomical relationships, the large adrenal mass altered the normal surgical anatomy [2,8,9]. Because of the large adrenal mass and the associated distortion of the regional vascular anatomy, major visceral arteries that would not ordinarily be encountered during routine left adrenalectomy, including the celiac artery and CMA, became incorporated into the operative field. Simultaneously, marked deformation of the phrenicoabdominal vein eliminated one of the principal anatomical landmarks routinely used during adrenalectomy, making reliable differentiation between expendable vessels and critical arterial structures impossible [8,9,10]. Consequently, the conventional technical advantage of the left-sided approach was effectively lost despite the absence of definitive vascular invasion. Reports specifically describing severe phrenicoabdominal vein deformation together with tumor-induced incorporation of the celiac artery and CMA into the operative field remain scarce in the veterinary literature. These findings suggest that operative complexity may be assessed not only according to tumor laterality or macroscopic invasion of major named vessels but also according to the degree of tumor-induced vascular distortion and loss of normal anatomical orientation [7,16,17].

Importantly, the principal surgical challenge in Case 2 resulted from profound tumor-induced vascular distortion rather than macroscopic invasion of the major named vessels. The resulting loss of normal anatomical orientation explained the inability to safely distinguish essential arteries from adjacent vascular structures during the initial exploratory laparotomy despite the absence of gross vascular invasion. Temporary discontinuation of the initial exploratory procedure should not be regarded as technical failure but rather as an appropriate intraoperative risk-mitigation strategy. Once the normal anatomical orientation could no longer be confidently maintained and critical arterial structures could not be reliably distinguished from the distorted regional vasculature, continued dissection was considered to carry an unacceptable risk of catastrophic vascular injury. Under such circumstances, staged surgical management, incorporating additional anatomical assessment and surgical replanning before definitive adrenalectomy, may facilitate safer surgical planning and reduce the risk of catastrophic vascular injury in anatomically complex adrenal tumors [18,19,20].

Although contrast-enhanced CT remains indispensable for preoperative evaluation of canine adrenal tumors, conventional multiplanar images may not fully represent the anatomical complexity encountered during surgery, particularly in cases with marked tumor-induced vascular distortion [7,16,17]. In Case 2, the combination of the large adrenal mass and severe displacement of the regional vasculature resulted in a limited operative field in which critical vascular structures could not be reliably identified. Advanced 3D vascular reconstruction therefore provided patient-specific anatomical information that directly influenced surgical planning [19,20,21]. Rather than serving solely as a visualization tool, the reconstructed images supported modification of the definitive surgical approach by adding a left paracostal incision, thereby improving surgical exposure of the distorted retroperitoneal anatomy [18,19,20]. These findings suggest that preoperative 3D vascular reconstruction may be particularly valuable in dogs with marked tumor-induced vascular displacement or loss of normal anatomical landmarks, especially when conventional CT fails to adequately define the relationship between the adrenal mass and adjacent major vessels [20,21].

The present cases demonstrate that surgical complexity in canine pheochromocytoma cannot be evaluated solely according to radiographic evidence of vascular invasion [16,17]. Rather, operative difficulty may arise through distinct mechanisms, including peri-vascular fibrous adhesion and tumor-induced distortion of the regional vascular anatomy, each of which may substantially influence surgical planning [2,7]. These findings emphasize the importance of comprehensive preoperative assessment that incorporates patient-specific anatomical characteristics in addition to vascular invasion and tumor laterality [8,9,21]. In anatomically complex cases, patient-specific anatomical information may facilitate refinement of the surgical approach and improve operative exposure [19,20,21]. Notably, unlike many previously reported cases emphasizing venotomy, thrombectomy, or vascular reconstruction for tumors with major vascular invasion, neither case required planned vascular lumen entry despite considerable operative difficulty [11,13,14,15]. Therefore, the anticipated need for advanced vascular procedures should not be regarded as the sole indicator of surgical complexity, which may instead be evaluated according to patient-specific anatomy, intraoperative findings, and the technical challenges anticipated during tumor dissection [2,8,9].

Several limitations of this report should be acknowledged. First, the precise mechanisms responsible for the intraoperative cardiovascular fluctuations observed in Case 2 could not be definitively established because multiple factors, including anesthetic management, tumor manipulation, and autonomic reflexes, may have contributed to the observed hemodynamic changes. Second, although 3D vascular reconstruction appeared to facilitate patient-specific surgical planning and modification of the operative approach in this case, the present report describes only two clinical cases and therefore does not establish objective criteria for identifying which adrenal tumors are most likely to benefit from this technique or support generalized recommendations regarding perioperative management. The surgical strategies described herein should instead be regarded as illustrative clinical observations. In addition, postoperative follow-up was conducted by telephone without repeat diagnostic imaging. Therefore, although no owner-reported clinical signs suggestive of recurrence or metastasis were identified during the available follow-up period, subclinical local recurrence or distant metastasis could not be excluded, particularly in Case 2, in which histopathological examination demonstrated malignant pheochromocytoma with tumor emboli. Further studies involving larger numbers of dogs are warranted to determine the anatomical or imaging characteristics that may justify selective use of 3D vascular reconstruction during preoperative planning. Third, although chronic kidney disease developed during long-term follow-up after unilateral ureteronephrectomy in Case 1, the present report cannot determine the relative contribution of nephrectomy, pre-existing subclinical renal disease, aging, or other patient-related factors to the subsequent deterioration in renal function. Therefore, the long-term renal consequences observed in this patient should be interpreted cautiously, and further studies are warranted to clarify the role of preoperative assessment of contralateral renal reserve when nephrectomy is anticipated during adrenalectomy. Nevertheless, the present cases provide clinically relevant illustrative observations regarding perioperative planning and intraoperative decision-making in dogs with anatomically complex pheochromocytomas and underscore the importance of evaluating surgical complexity beyond macroscopic invasion of major named vessels alone.

## 4. Conclusions

The present report demonstrates that surgical complexity in canine pheochromocytoma is not determined solely by macroscopic invasion of major named vessels but may also result from extensive peri-vascular adhesion and profound tumor-induced vascular distortion. Progressive distortion of normal vascular landmarks may substantially increase the difficulty of adrenalectomy, even for left-sided adrenal masses that are traditionally considered less technically demanding. These cases further highlight the importance of patient-specific anatomical assessment and individualized perioperative planning, as operative strategies may be tailored according to the underlying mechanism of surgical complexity rather than the presence or absence of macroscopic invasion of the major named vessels alone. Advanced 3D vascular reconstruction may facilitate patient-specific surgical planning when conventional CT fails to adequately characterize complex vascular anatomy, while flexible intraoperative decision-making and staged surgical management may further improve operative safety in anatomically challenging cases. Importantly, these cases further demonstrate that technically demanding adrenalectomy may occur even without planned venotomy or vascular reconstruction when severe peri-vascular adhesion or tumor-induced anatomical distortion is present.

## Data Availability

The raw data supporting the conclusions of this article will be made available by the authors on request.

## Abbreviations

The following abbreviations are used in this manuscript:

3D	Three-dimensional
ACTH	Adrenocorticotropic hormone
BUN	Blood urea nitrogen
CdVC	Caudal vena cava
CMA	Cranial mesenteric artery
CRI	Continuous-rate infusion
CT	Computed tomography
IV	Intravenous
LC-MS/MS	Liquid chromatography–tandem mass spectrometry
NMN:Cr	Urinary normetanephrine-to-creatinine ratio
PCV	Packed cell volume
PO	Per os (oral administration)
SAP	Systolic arterial pressure
SC	Subcutaneous
UCCR	Urine corticoid-to-creatinine ratio
